# Cigarettes and the Boys

**Published:** 1906-07

**Authors:** 


### EDITORIAL NOTES AND COMMENTS.

**The Business office of THE JOURNAL-RECORD is No. 1007-1008 Century Building.**

**The Editorial office is 318-319-320 The Prudential.**

**Address all Business communications to Dr. M. B. Hutchins, Mgr.**

**Make remittances payable to THE ATLANTA JOURNAL-RECORD OF MEDICINE.**

**On matters pertaining to the Editorial and Original Communications address Dr. Bernard Wolff, Atlanta.**

**Reprints of original articles will be furnished at cost price. Requests for the same should always be made *on the manuscript*.**

**We will present, post-paid, on request, to each contributor of an original article, twenty (20) marked copies of THE JOURNAL-RECORD containing such article.**

### CIGARETTES AND THE BOYS.

TO-DAY the cigarette is looked upon by all smokers as the very worst form of tobacco addiction. It is so cheap, it is so generally inhaled, it is often so loaded with drugs, and so many times made up from cigar stumps and street scrapings, that there is every argument against its use.

Rev. James L. Hammond tells what he saw in a San Francisco opium den: "In a room not more than twenty feet square, down three stories underground, dimly lighted and dingy, where the air was so foul it almost overcame you as you stood in the entrance, I found twelve Chinamen, busy at work. Sitting flat on the floor, in the midst of indescribable filth, they were rolling cigarettes for the American boy to smoke.

"There was a great pile of material in one corner of the room, and we struck a match and stooped down to examine it. We found it was cigar stumps and quids of tobacco, mixed with the vilest of sewer excretions."—*The Healthy Home.*

The foregoing is given as typical of the variety of argument advanced against the cigarette habit. That it is based upon a sincere desire to better humanity by eliminating its weaknesses and indulgences, no one will doubt, but there is fair room for argument as to how far puerile misrepresentation and palpable misstatement will effect this laudable purpose. There are many of these self-elected reformers who look at everything which does not coincide with their philosophy of life and their interpretation of its moral responsibility with a jaundiced eye and leave no style of argument, even the most vituperative abuse unexploited, to arouse public sentiment against the alleged delinquencies. They do not know what temperate discussion is nor decent controversy, or at least they do not practice either. Abuse does not constitute argument, it rather weakens it, as it is practically an acknowledgment that the search after truth—the solution of all problems—has been abandoned and unconvincing and unilluminating personalities substituted. In order to ascertain the exact status of a thing it is necessary that one must judge and conclude from known facts, not unsubstantiated belief. There is a world of difference between that which is known to be true and that which is merely believed to be true, but which in reality may not be true at all. Now, in the present instance, is it known and forever established that cigarette-smoking is acknowledged by all smokers to be the worst form of tobacco addiction. Is not this statement just an easy assumption of finality that suits the purposes of the good but rather flatulent moralists? The articles which appear in scientific publications from time to time, based upon chemical analysis and clinical observation of the cigarette and its users, go to show that this form of tobacco addiction is not only the worst but is perhaps the least harmful. One by one the popular delusions concerning the cigarette have been shown to be fallacious. Some years ago William Lloyd Garrison (not he of anti-slavery fame, but a much more liberal-minded bearer of the same name) wrote what he called a Brief for the Cigarette. Among other things he showed so plainly that none but a general agnostic or a maul-headed fool could see that the alarming stories of insanity due to cigarette-smoking were newspaper exaggerations and fakes pure and simple, and that no psychiatrist in charge of any of the insane asylums regarded cigarette addiction as a factor in the production of feeble-mindedness or insanity. It was present among many mentally defective, but was in no wise responsible for its existence. The current assertion by the unfriendly that opium is contained in cigarettes is likewise emasculated by the fact that the cheapest form of opium costs about $15 a pound, and by a very simple rule in economics, one does not sell his goods at less than cost.

The attractive picture of the Chinese rolling cigarettes in a noisome den is more picturesque than convincing. The product of their thrift and industry, while it might contain cigar butts, dried, and cold quids and "sewer excretions," must have been intended for their own consumption. The Chink's well-known dietary liberality and discursiveness, running all the way from dried cliff-swallow saliva, through shark's fins to puppies, would pave the way for the belief that a pinch of the scraping from a catch basin might even add a certain piquancy to their smoke when mixed with due proportions of "snipes" and "dead soldiers." Who knows?

But that the olfactory and gustatory senses of the American boy will be deceived by such gross tricks of substitution does not immediately appeal to the reason. For the sake of clearness, it would be pertinent to enquire what sewer excretions are. The sewer, as a sanitary adjunct, serves an unesthetic but highly effective purpose, but to say that it is an organ of excretion rather overstates the case. It gives rise in hot, dry weather to divers odors, savors and stenches, but for these it is in no wise responsible. It does not secrete them; it merely passively allows them to pass or not to pass, according to the state of its permeability.

Until the American Tobacco Company, which, it is generally understood, controls the cigarette output, by an *a la mode* inquiry in its affairs is forced to confess that the popular brands are made in cellars by Chinese and are composed of cigar stumps, quids and sewer excretions, the great procession of cigarette-smokers will be more than likely, despite these alarming disclosures, to jog along in their old *insouciant* faith in Bull Durham and Duke's Mixture. At any rate, there is a chance that sewer excretions will be only an occasional seasoning, and we are willing to take that chance among the goods the gods provide. If the true friends of man wish to shoot folly as she flies, they must load their shells with something more effective than wormy peas to bring her down.

